# Social status and personality: stability in social state can promote consistency of behavioural responses

**DOI:** 10.1098/rspb.2013.2531

**Published:** 2014-01-07

**Authors:** Anna Favati, Olof Leimar, Tommy Radesäter, Hanne Løvlie

**Affiliations:** 1 Department of Zoology, Stockholm University, 106 91 Stockholm, Sweden; 2 Department of Physics, Chemistry and Biology, Linköping University, 581 83 Linköping, Sweden

**Keywords:** behavioural syndromes, intra-sexual selection, phenotypic plasticity, social dominance, chicken

## Abstract

Stability of ‘state’ has been suggested as an underlying factor explaining behavioural stability and animal personality (i.e. variation among, and consistency within individuals in behavioural responses), but the possibility that stable social relationships represent such states remains unexplored. Here, we investigated the influence of social status on the expression and consistency of behaviours by experimentally changing social status between repeated personality assays. We used male domestic fowl (*Gallus gallus domesticus*), a social species that forms relatively stable dominance hierarchies, and showed that behavioural responses were strongly affected by social status, but also by individual characteristics. The level of vigilance, activity and exploration changed with social status, whereas boldness appeared as a stable individual property, independent of status. Furthermore, variation in vocalization predicted future social status, indicating that individual behaviours can both be a predictor and a consequence of social status, depending on the aspect in focus. Our results illustrate that social states contribute to both variation and stability in behavioural responses, and should therefore be taken into account when investigating and interpreting variation in personality.

## 1. Introduction

Understanding variation in phenotypes is a key issue in evolutionary biology [1]. The topic includes the study of animal personality, in the form of consistent individual differences in behaviour, across time and/or situations [2], also referred to as temperaments [3] or coping styles [4]. The phenomenon has been described in a large number of species in multiple taxa [5], but there are still major gaps in our understanding of why there is personality variation, including unanswered questions about the mechanisms behind stable behavioural responses and the evolution and maintenance of behavioural polymorphism [6,7]. Properties of an individual or circumstances that may affect the costs and benefits of its behaviours, such as size or energy reserves, are sometimes referred to as ‘states’ [8]. While the cause of this variation may or may not be known, stability in these states is theoretically predicted to produce stability in behavioural responses which, in combination with between-individual variation in state, gives rise to variation in behavioural types, and thus personality [2,6,9]. More broadly, any state changing more slowly than behaviour *per se* is predicted to cause short-term stability in state-dependent behavioural responses [2]. Together with a positive feedback system, stability in state may also generate long-term stability of behavioural types [9]. Social relationships such as pair bonds or status hierarchies could be examples of such states. If social positions or relationships constitute stable states, then intra-individual stability of behavioural responses would follow as a consequence, whereas the behavioural responses should change when the social state of an individual is changed. Despite the intuitiveness of these predictions, the importance of social states for personality variation has not yet, to our knowledge, been empirically tested.

In social species, social relationships often take the form of dominance hierarchies, which, in turn, are based on repeated outcomes in favour of one participant of dyadic agonistic interactions [10]. Socially dominant individuals commonly enjoy increased access to resources, such as mating partners, which typically results in a positive relationship between social status and reproductive success [11,12]. Aggression and the ability to dominate conspecifics can correlate positively with boldness, exploration and active stress handling, thereby defining a ‘proactive’ behavioural style of the reactive–proactive coping style continuum [4,13]. For example, explorative great tits (*Parus major*) win more fights compared with less explorative ones [14], and bold three-spined sticklebacks (*Gasterosteus aculeatus*) are more aggressive than shyer individuals [15]. On the other hand, boldness and exploration have been found to be unrelated, or negatively correlated, with social dominance [16,17]. This indicates that the relationship between personality and social status can be species-specific, but also that there are currently limitations to our understanding of the relationship between them.

In principle, there are three possible scenarios for observed correlations between social dominance and personality traits. First, different social positions can be associated with different behavioural tendencies [18]. These differences can also manifest themselves outside the social group, and thus influence responses in personality assays [19]. In this scenario, it is expected that behavioural responses are flexible, adjusting to the current social position, at least to some extent. Second, differences in behaviour can directly influence the chance of obtaining a certain social position (e.g. aggression [20]). In this scenario, certain personality types are more likely to be found in specific social positions. However, and crucially, behavioural responses are independent of experimental change of social positions. Third (and partially overlapping with the previous), behaviour and social position can have a common underlying cause [13,21]. In such a case, both personality traits and the underlying cause (e.g. hormonal state [21]) might predict social position, but behaviour is not necessarily altered when the social position is changed. Although there is some support for each of the three scenarios separately, they have not previously been tested simultaneously, leaving the causality between behaviour and social status unclear. Our aim here is therefore to investigate the issue by means of an experimental approach where social status of individual male domesticated fowl (*G. g. domesticus*) is changed and behaviour scored in personality assays. If a change in dominance status leads to a change in behavioural response, then we can conclude that social status may represent a state that gives rise to consistent differences in behaviour.

Male fowl are suitable for investigating the potential link between variation in personality and social status for two main reasons. First, dominance hierarchies are relatively stable; the status of an individual persists for approximately one breeding season in free-ranging flocks of fowl [22], but changes in group composition over shorter time spans can occur and cause changes in the status of individuals (e.g. if the dominant male is predated [23]). Therefore, status can be considered as a slowly changing, yet not permanent state. Second, dominant and subordinate male fowl differ quantitatively in behaviour. For example, dominant males crow more, as a signal of territoriality, and spend more time being vigilant compared with subordinate males [18,24]. Nevertheless, the causal relationship between behavioural differences and status warrants further investigation.

## 2. Methods

### (a) Animals and housing

The study took place during the breeding seasons (May–September) of 2007 and 2011 at Tovetorp Research Station, Stockholm University, Sweden. We used 84 males (2007: *n* = 36; 2011: *n* = 48) of an old Swedish game breed of fowl (*G. g. domesticus*, ‘Gammal svensk dvärghöna’) used to human handling and kept under semi-natural conditions in mixed-sex, mixed-age (1–9 years) groups (10–18 individuals per group). Data were collected during the hours of day when the birds are most active (05.30–11.00 and 15.00–19.00 h local time, see Løvlie & Pizzari [25] for further information on variation in daily behavioural patterns). Groups of four males (*n*_groups_ = 21) were matched for morphological measures (less than 10% difference in comb and body size) to reduce the effect these variables can have on the establishment of status [20,26,27]. Comb size and body size were estimated as comb length and tarsus length, respectively, measured with digital callipers to the nearest tenth of a millimetre. Body size was also estimated as body weight measured with a digital scale to the nearest gram. To generate males of different social positions, two pairs of males were randomly chosen from each of the matched groups of four males (*n*_pairs_ = 42). Pairs of males were housed in outdoor aviaries (approx. 3 × 3 m) that were visually, but not vocally, isolated from other birds. This set-up resulted in one of the males in each pair becoming dominant, and the other subordinate. A minimum of five observed successive submissive behaviours (i.e. avoiding the other male when he approached) by a male within 2 h, defined that male as subordinate [28]. Males within a pair had not been housed together for at least two weeks prior to the trials, reducing any effects earlier encounters may have had on the establishment of social status [29,30]. All birds had ad libitum access to food and water, and all aviaries had dust baths and perches.

### (b) Behavioural assays and manipulations of social status

Two days after the pair was formed, which was enough time for males to establish a dominance relationship without any reversals, each bird was exposed to its first ‘novel arena’ test (referred to as ‘trial 1’) in a 3×6 m large outdoor area connected to the aviary by a door (see the electronic supplementary material, figure S1). The order males were tested in a pair was random with respect to social dominance. The floor in the arena was covered with dark brown peat to create novelty (in comparison with the sandy floor of the birds’ home pens) and divided into eight subareas by subtle lines drawn in the floor substrate to facilitate estimates of exploration (see below). The arena had five artificial bushes (green plastic spruce trees, approx. 70×50 cm) in order to obstruct direct view of the arena, and thus encourage exploration (see the electronic supplementary material, figure S1). The cage mate of the focal male was removed from the aviary by herding him into an empty adjacent pen 2 min before the test started. The door between the aviary and the test arena was opened, and focal males that entered the arena within 30 s were considered bold, whereas those who did not were considered shy (i.e. a ‘boldness’ spectrum was split into two categories because of the clear bimodal distribution of the latencies). In cases when a male did not voluntarily enter the arena within 2 min, he was gently herded into it by the observer. The observer sat outside the arena, visible to the male during the test. Behaviours and vocalizations were recorded for 15 min, starting when the male entered the arena. Vigilance (head above shoulder height, eyes open) and behaviours categorized as non-vigilance (eating, preening, resting and dust bathing, behaviours that all occurred in lower frequencies) were recorded every 30 s, and the proportion of recordings a male was vigilant during the test was calculated (hereafter named ‘vigilance’). The number of unique subareas a male visited (1–8) was used as a measure of ‘exploration’ propensity. The number of times a male entered a subarea he had previously visited was noted as a measure of ‘activity’. Number of crows was recorded ad libitum (‘crowing’). Crowing is a species-specific vocalization related to territoriality in male fowl [31]. In addition to crowing, some males uttered alarm calls, which are loud and distinct warning vocalizations typically uttered when an individual is startled, for example when a predator appears [32], but these few vocalizations were not analysed further.

When the behaviour of all four males of a matched group had been scored in the first novel arena test, pair members were experimentally changed, so that the two previously dominant males formed a new pair, and the two subordinate males another pair. The new pair set-up forced one male in each pair to change social status, as there can only be one dominant male and one subordinate male in each pair (see [18] for previous use of this design). After 2 days of acclimation to the new social context, the novel arena test was performed a second time in order to estimate the influence of changes in social status on behaviour [27]. We refer to this test as ‘trial 2’. In a strict sense, the arena was not novel to the males during the second trial. However, the mean values for each behavioural response were similar in trial 1 and trial 2 (see the electronic supplementary material, table S1), suggesting that the behaviour was not greatly affected by habituation to the arena. For the second pair set-up, the number of males was reduced to 82 owing to the death of one male in 2007 and injury of one male in 2011. A.F. conducted all observations.

### (c) Statistical analyses

Our general approach to the statistical modelling of the behavioural responses was to first determine suitable Box–Cox transformations of the continuously distributed responses, in order to achieve homogeneous variances. The outcome of this procedure was that the measure vigilance did not need transformation, the transformation (*x* + 1)^0.25^ was used for activity and crowing, and the transformation *x*^3^ for exploration. We applied these transformations in all analyses. The binary measure of boldness was treated as a discrete variable during analysis, and is presented in the figures as the proportion of males that were bold. Based on our experimental design (see §2*a*), with males being divided into matched ‘groups’ (21 groups of four males), we used the ‘group’ as a source of random effects in mixed models of the behavioural responses. The relationships between response variables were also explored by Spearman rank correlations conducted separately for males that either changed or remained in the same social position during the two pair set-ups (see the electronic supplementary material, table S2).

#### (i) Difference in behavioural responses of dominant and subordinate males prior to changed status

To investigate differences in behavioural responses between dominant and subordinate males (*n* = 84), we analysed the effect of social status on each response (boldness, vigilance, activity, exploration and crowing) in trial 1 (i.e. prior to experimentally manipulated social status), by fitting mixed models with the trial 1 social status as a fixed effect and the group as a random effect (Bayesian Markov chain Monte Carlo generalized linear-mixed models, MCMCglmm; see the electronic supplementary material for further details of the MCMCglmm procedures [33]).

#### (ii) Individual characteristics versus the effect of social status on behavioural responses

To investigate the influence of social status on behavioural responses after social status had been experimentally manipulated, behavioural responses recorded in trial 2 were analysed further. The behavioural response in the second trial can show statistical covariation with the behavioural response in the first trial (indicating an effect of individual characteristics, beyond social status), current social status (showing that behaviour is a plastic response to the social state) or the previous social status (suggesting a common underlying variable for behaviour and social status). In order to disentangle the effects of social status and individual characteristics on a behavioural response, we fitted statistical models of the effect of ‘current status’ (obtained in the second pair set-up, ‘dominant’ versus ‘subordinate’), ‘previous status’ (obtained in the first pair set-up, ‘dominant’ versus ‘subordinate’), and the behavioural response shown in the first trial on each of the responses in the second trial. The statistical significances of these effects were determined using Bayesian MCMCglmm analysis. In these analyses, we used the group (of four matched males) as a random effect and, if this improved the fit, also the interaction between group and the first trial response as an additional random effect. In these analyses, we examined the robustness of the MCMC runs by trying out different values for burn-in periods and thinning intervals, as well as examining autocorrelation plots (see the electronic supplementary material for further details).

#### (iii) Factors predicting social status

Comb size, body size (tarsus length and body weight) and age are traits that may affect the establishment of status in male fowl [20]. We investigated whether the initial matching of males for these properties was successful in both pair set-ups by testing for the difference in these traits between dominant and subordinate males in each pair by means of paired *t*-tests. The level of this analysis was thus the pair (*n*_trial1_ = 42, *n*_trial2_ = 40). To investigate whether behaviour may predict social status, we tested whether behavioural responses in the first novel arena test could predict the outcome of the duel in the second pair set-up. Difference between dominant and subordinate males in the binary response boldness was tested with Fisher's exact test, whereas difference in vigilance, activity, exploration and crowing between the dominant and subordinate male of each pair, were tested with a paired *t*-test (*n*_pairs_ = 41).

All statistics analyses were conducted using R v. 2.15.1 [34], and the package ‘MCMCglmm’ [33].

## 3. Results

### (a) Differences in behavioural responses of dominant and subordinate males

**Figure 1.**
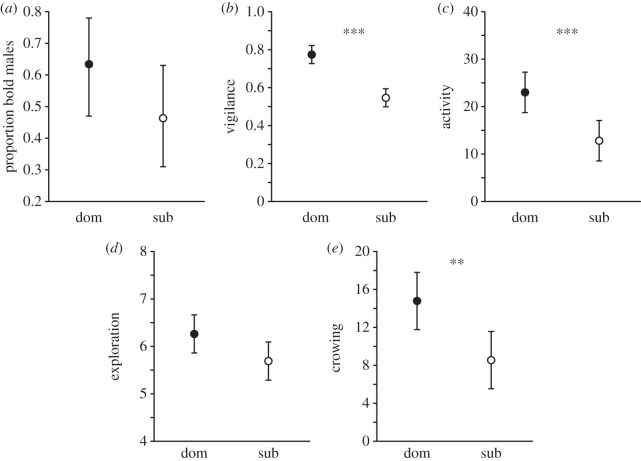
Behavioural response of dominant and subordinate males in the first trial of a novel arena test. Dominant males (‘dom’, filled circles) (*a*) tended to be bolder (i.e. more often entered the arena within 30 s), (*b*) were more vigilant (i.e. had higher frequency of time spent being vigilant), (*c*) were more active (i.e. performed more subarea transitions), (*d*) tended to be more explorative (i.e. visited more subareas), and (*e*) crowed more often (i.e. number of crows) compared with subordinate males (‘sub’, open circles; table 1). Values are given as untransformed mean±95% confidence interval (CI), except for (*a*), where the proportion of bold males±95% CI, are given.

Dominant males were more vigilant and active, and crowed more compared with subordinate males, but did not differ significantly from subordinates in boldness and exploration (data from first trial: table 1 and figure 1; see also the electronic supplementary material, table S3 for estimates of the random effects, that were all fairly small).

**Table 1.** Behavioural responses of dominant and subordinate male fowl in the first trial of a novel arena test. (Values presented are mean±s.e. for all responses except boldness, where the proportions of bold males are shown. *P*_MCMC_-values < 0.05 are highlighted in italics.)

response	dominant	subordinate	*P*_MCMC_
boldness	0.64	0.45	0.060
vigilance	0.79 ± 0.02	0.55 ± 0.03	*<0.001*
activity	23.00 ± 2.36	12.81 ± 1.97	*<0.001*
exploration	7.26 ± 0.17	6.69 ± 0.24	0.066
crowing	14.79 ± 1.56	8.55 ± 1.52	*<0.001*

### (b) Individual characteristics versus the effect of social status on behavioural responses

**Figure 2.**
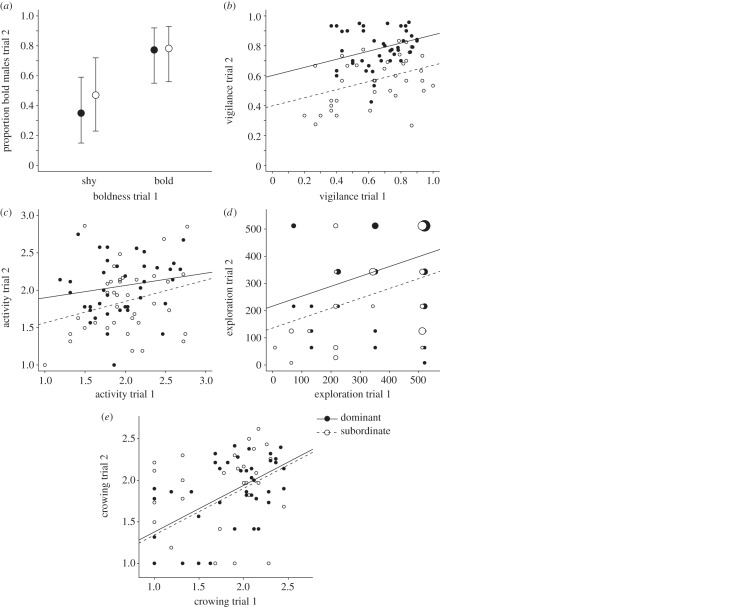
The effect of current social status (in trial 2) and previous behavioural responses (in the first novel arena test, i.e. *‘*trial 1’) on the responses of male fowl in a novel arena test after social status was manipulated (i.e. ‘trial 2’). (*a*) Status in trial 2 (dominant males: filled circles, subdominant males: open circles) did not affect the proportion of males that were bold (i.e. males that entered the arena within 30 s), but previous boldness (*x*-axis) had a significant effect on boldness in trial 2 (*y*-axis). Males that were dominant in trial 2 (filled circles, solid line) were (*b*) more vigilant (i.e. had a higher frequency of time spent being vigilant), (*c*) more active (i.e. conducted more subarea transitions), and (*d*) more explorative (i.e. visited more subareas) compared with males that were subdominant (open circles, dashed line). At the same time, these behaviours were repeatable within individuals (demonstrated by a positive slope; table 2). (*e*) Social status in trial 2 did not affect crowing. The figures show transformed response variables (for transformations, see main text).

**Figure 3.**
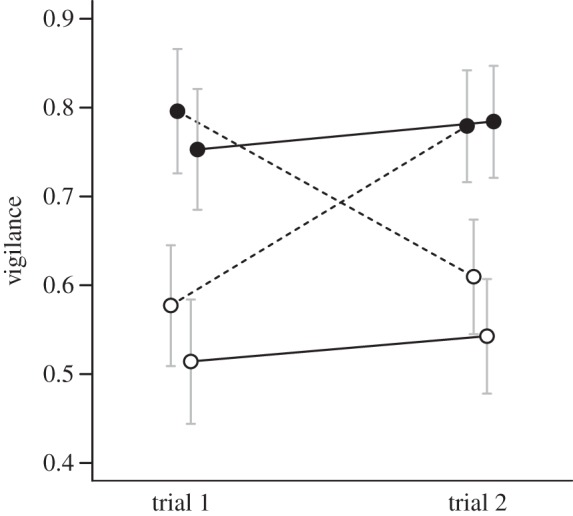
Vigilance of male fowl of different social status during the first (trial 1) and second test trial (trial 2) in a repeated novel arena test. Dominant males (filled circles) were more vigilant (i.e. frequency of time spent being vigilant) compared with subordinate males (open circles). Current social status affected this behaviour, such that males who changed social status also changed the time spent being vigilant (dashed lines), whereas males with the same status during both trials were more consistent in their frequency of vigilance. Values presented are mean ± 95% CI. See the electronic supplementary material for similar figures for other behaviours (figure S2).

Our analyses of factors affecting the observed behavioural responses of males after social status was manipulated are presented in table 2. Table 2 shows, for each response from the second trial (i.e. ‘trial 2’), the estimated model parameters and the statistical significances obtained. As an example, for vigilance in trial 2, table 2 shows that vigilance in trial 1 entered into the model with a coefficient of 0.32, showing that there was positive covariation between the vigilance responses in the two trials. Further, the status of a male in trial 1 did not have a significant effect on his vigilance in the second trial, but there was a significant effect of status in the second trial, such that males dominant in the second trial had 0.20 higher vigilance than subordinate males in trial 2. The results for the other trial 2 responses in table 2 are interpreted in a similar way (see also the electronic supplementary material, table S4 for the estimates of the random effects). These analyses show that all trial 2 response variables investigated had a statistically significant positive relationship with the corresponding response in the first trial (table 2), in other words showing intra-individual consistency. At the same time, all responses but boldness and crowing were affected by the current social position of a male (table 2). Vigilance, activity and exploration were flexible behaviours and changed to a large extent with changes in social status (table 2 and figure 2*b–d*, also illustrated by figure 3 showing variation in vigilance; for similar figures for other variables, see the electronic supplementary material, figure S2). This was especially pronounced for vigilance (figures 2*b* and 3), whereas, for example, boldness did not follow changes in status (figure 2*a*). Males that had changed status clearly altered the time they spent being vigilant: males with reduced status from dominant to subordinate showed a decrease in vigilance, whereas an increase in vigilance was seen in males that improved their status from subordinate to dominant (figures 2*b* and 3). Males with stable status across the two experimental pair set-ups (males that remained either dominant, or subordinate) showed a stable between-test response (figures 2*b* and 3). The number of crows uttered by males was dependent on the previous social status: males that previously had been dominant crowed more in the second trial compared with other males, irrespective of their current social status (table 2 and figure 2*e*).

**Table 2.** Factors explaining variation in behavioural responses of male fowl in the second trial (i.e. after social status was manipulated) of a novel arena test. Values presented are posterior means of model parameters from the MCMCglmm analyses, with effects of status given as dominant-subordinate differences (i.e. positive values indicates that dominant males had a higher value). See main text for variable transformations used. (The asterisk symbols indicate statistical significance: *P_MCMC_ < 0.05, **P_MCMC_ < 0.01, ***P_MCMC_ < 0.001.)

response trial 2	response trial 1	status trial 1	status trial 2
boldness	2.13***	−0.42	−0.36
vigilance	0.32***	−0.04	0.20***
activity	0.30*	−0.23**	0.21*
exploration	0.37**	0.30	78.68*
crowing	0.44***	0.36***	0.08

### (c) Factors predicting social status

None of the measured morphological traits, or age had a detectable effect on the establishment of social status in this experiment, indicating that the experimental matching for these features was successful (first pair set-up: tarsus length, *t*_41_ = −0.080, *p* = 0.94; body weight, *t*_41_ = 0.36, *p* = 0.72; comb length, *t*_41_ = −1.03, *p* = 0.31; age, *t*_41_ = 0.49, *p* = 0.63, second pair set-up: tarsus length, *t*_40_ = 0.76, *p* = 0.45; body weight, *t*_40_ = 0.0072, *p* = 0.99; comb length, *t*_40_ = 1.71, *p* = 0.09; age, *t*_40_ = −0.14, *p* = 0.89). On the other hand, the number of crows a male uttered in the first trial predicted future social status in the second pair set-up; males that crowed more had a higher probability of becoming dominant compared with males that crowed less (*t*_40_ = 2.59, *p* = 0.01). No other behavioural responses predicted future status (boldness, *p* = 0.58; vigilance, *t*_40_ = 0.41, *p* = 0.68; activity, *t*_40_ = −0.73, *p* = 0.47; exploration, *t*_40_ = 0.78, *p* = 0.44).

## 4. Discussion

Our experiment showed that behavioural responses of male domestic fowl in behavioural assays are affected both by the social status of an individual and by its individual characteristics. Our results support the general interpretation of behaviours being plastic but also showing constraints in flexibility, in line with the few empirical studies where both possibilities have been investigated [35,36]. How social position and individual characteristics influence behaviour depends on the behavioural response in focus, and will be discussed below.

We introduced three scenarios for how a relationship between social position and behavioural responses can come about. By exploring different aspects of behaviour of male fowl across changes in social position, we found experimental support for two of the three scenarios, namely that the social position in itself influences behaviour, and that social position and behaviour may share a common, underlying cause. According to the first of these, frequencies of behaviours are expected to change when social status changes, thereby setting a limit to the consistency of the individual responses. We found this to be the case for three of the recorded responses: activity, exploration and vigilance. Activity and exploration are commonly used behavioural dimensions in personality studies [3], but the effect of social status on their expression has previously, to our knowledge, not been investigated. In the fowl, dominant males guard territories [31]. The increased activity and exploration observed when a male becomes dominant may thus be explained by an increased propensity to patrol a territory and its surroundings. Our results support the general idea that individuals obtaining dominant social positions are more often observed to behave in a proactive way, rather than in a reactive manner [4,14,21]. They further suggest that an individual's degree of activity and exploration is partly a consequence of the current social situation, and stays constant if social status is unchanged, but changes when social status changes. This is in line with the reasoning of Dall *et al*. [2], who suggested that stability in various states can underlie consistency in behavioural responses.

Vigilance decreases predation risk by facilitating early detection of predators and can be considered a response to an individual's assessment of risks in its environment [37]. Both in our study and as previously shown [18], dominant males were more vigilant, and the degree of vigilance changed dramatically when status changed, which can be interpreted as a risk-averse response to protect increased assets (e.g. access to females as sexual partners) [6]. Such a response follows the principle of asset protection, in that individuals should increase their cautiousness when assets increase [38,39]. However, it has been argued that differences in state should converge over time, as cautiousness is assumed to lead to a decrease of assets [9]. We do, however, not expect increased access to females—as a consequence of high social status—to be negatively affected by an increased vigilance, thus the relationship between asset and behaviour in this case need not function as a negative feedback mechanism that causes a convergence of individual states. Our results indicate that social status could act as a stabilizing condition, or ‘state’, generating at least short-term stability in behavioural responses [39,40]. Whether long-term stability is achievable in a similar way remains to be studied. This could happen if, for example, early experiences of dominance positions lead to long-term individual differences in physiology or morphology that later in life affect the chances of obtaining a certain social position.

We also found support for the scenario that behaviour and social dominance share common underlying causes. The number of crows uttered by a male in the final novel arena test was correlated with both the previous social status and the crowing in the previous novel arena test, indicating that individual males varied in their underlying propensity to crow and that this propensity also influenced the chances of becoming dominant. The observation that dominant males crow more than subordinates has been reported previously in the species [18,24], but to the best of our knowledge this study is the first observation of a correlation between social position and the number of crows uttered outside of a social context. In addition, we found that when two dominant, or two subordinate males, were paired up in preparation for the second trial, the male that had crowed more in the preceding novel arena test more often became dominant. Our observations thus suggest that crowing frequency partly reflects an underlying characteristic that influences the establishment of social status [21,41,42]. Note, however, that crowing is not a commonly observed behaviour during the interactions that determine social status; it rather appears as a signal of the outcome [24,43]. A potential underlying factor for crowing and social dominance could be testosterone, which regulates development of crowing frequency in male fowl [44]. A higher testosterone level during male–male interaction has been found to be associated to shorter attack latencies and an increase in the probability of obtaining high social status in male domestic fowl [45]. Further studies of the relationship between crowing, behaviour during duels and hormonal status could help elucidate why crowing in a non-social situation not only signals high social status, but also predicts it.

Our experimental design did not allow an explicit and thorough test of the remaining scenario; that behaviour *per se* leads to a certain social position, because males in our study had already obtained a social position prior to the first novel arena test. Behavioural responses from personality assays have sometimes proved to be predictive of future social status [4,14], but that possibility is not mutually exclusive with the two other scenarios presented here, and thus remains to be investigated further.

Of the responses studied here, only the level of boldness was unaffected by changes in social status. Dominant males tended to be bolder than subordinate males in the first trial, but there was no change in boldness when status was changed. Compared with the other behaviours we investigated, boldness therefore appears to be less flexible, in being independent of changes in social status. However, a recent study of sea anemones (*Actinia equina* [46]) showed that the experience of a fight, and in particular for the losing part, decreases boldness (latency to retain regular activity after being startled). This indicates that the social environment can affect also boldness.

In addition to the sometimes striking effect of social status on behavioural responses in our study, there was always a remaining correlation between the responses of individuals across the two test occasions. This consistency in behaviour across social situations is an expression of individual characteristics that fall under the general heading of animal personality, but the precise nature and general significance of these characteristics is an open question. Studies on other species have shown that heritable components, together with early life experiences (e.g. maternal effects), can partly explain variation and stability of individual behaviour [47,48]. Less attention has been directed to how intrinsic or environmental factors later in life may shape personality. An recent experimental study on great tits (*P. major*) showed that breeding effort is a potential example of a non-genetic cause of personality variation [49], in a broadly similar way as we found here for social positions. Our results emphasize the need for controlling for stability in the social context that is of importance for the species in question (e.g. social status, group composition), and possibly stability also in other contexts or dynamic states, to further improve our understanding of causes and consequences of variation in personality.

## Notes

Experiments were conducted according to ethical requirements in Sweden (Linköping Ethical committee, ethical permit no. 60-10).

## Data accessibility

Data deposited in the Dryad Repository (www.datadryad.org): http://doi.org/227qn.
